# Supplementary material for: Content-rich biological network constructed by mining PubMed abstracts
Source: BMC Bioinformatics. 2004 Oct 8;5:147. doi: 10.1186/1471-2105-5-147 (PMC528731; doi:10.1186/1471-2105-5-147)
Supplement: Additional File 2 — The original results of the above study (non-essential files are deleted to keep the file size under the limit set by BMC bioinformatics). [file 1471-2105-5-147-S2.bz2 › chilibotAdditionalFile2/dip10/727ID9822594E18076/html/HLYB_HLYA.html]

 


 **HLYB** and **HLYA** 
  
Found 91 abstracts in PubMed, retrieved 10.  
 

 What does Google say? 
 PDF only 
| .edu only 

---

**Interactive relationship** (e.g. stimulation, inhibition, etc)

**Neutral relationship**- Our data suggest that TnhlyAs generated hemolysin  [ **HLYA** ]  fusion proteins are secreted efficiently by the  **HlyB**  HlyD TolC hemolysin  [ **HLYA** ]  secretion machinery.  Ref: 9711856 FEMS Microbiol Lett, 1998
- The mutant  **HlyB**  HlyDDelta45 inner membrane complex engaged the hemolysin  [ **HLYA** ]  substrate, but this substrate engaged complex failed to trigger recruitment of TolC.  Ref: 11676535 J Mol Biol, 2001
- We show that an scFv  **HlyA**  hybrid secreted by Hly type I transporter TolC,  **HlyB** , HlyD is accumulated in the extracellular medium with the disulphide bonds correctly formed.  Ref: 11309117 Mol Microbiol, 2001
- The export of Escherichia coli hemolysin  [ **HLYA** ]  across the cytoplasmic and the outer membranes requires the COOH terminal signal sequence of  **HlyA** , the two specific translocator proteins  **HlyB**  and HlyD, and the outer membrane protein TolC.  Ref: 9630987 Nat Biotechnol, 1996

**Non-interactive relationship** (e.g. studied together, co-existance, homology, etc.)

- Mutant  **HlyB**  that bound, but did not hydrolyse ATP, supported IM complex assembly, substrate recruitment and bridging, but  **HlyA**  stalled in the channel.  Ref: 9822594 EMBO J, 1998
- The structure and function of the components of the  **HlyA**  secretion apparatus,  **HlyB** , HlyD and TolC, have been studied in great detail.  Ref: 11755084 Trends Microbiol, 2002
- The presence of fragments of genomes  **hlyA** ,  **hlyB** , papAH, papC, sfaG, sfaA and kps MT, associated with the pathogenicity islands of Escherichia coli, in clinical strains of other genera of the family Enterobacteriaceae, has been experimentally evaluated with the use of PCR.  Ref: 12506618 Zh Mikrobiol Epidemiol Immunobiol< MedlineTA>Zh Mikrobiol Epidemiol Immunobiol, 2002
- The toxin  **HlyA**  is exported from Escherichia coli, without a periplasmic intermediate, by a type I system comprising an energized inner membrane IM translocase of two proteins, HlyD and the traffic ATPase  **HlyB** , and the outer membrane OM porin like TolC.  Ref: 9822594 EMBO J, 1998
- Taking these results together, we propose a model for an early stage of initiation of secretion in vivo, in which the NBD of  **HlyB** , specifically recognizes the C terminus of the transport substrate,  **HlyA** , and where secretion is initiated by subsequent displacement of  **HlyA**  from  **HlyB**  by ATP.  Ref: 12662939 J Mol Biol, 2003
- We have purified the nucleotide binding domain of  **HlyB**   **HlyB**  NBD and a C terminal 23kDa fragment of  **HlyA**  plus the His tag HlyA1, which contains the secretion sequence.  Ref: 12662939 J Mol Biol, 2003
- We have developed an export cloning system that is composed of two vectors one in which the fusion of the desired gene with the 3 end of  **hlyA**  is generated, and a second in which the sequences containing the fusion are combined with the accessory genes  **hlyB**  and hlyD, thereby reconstructing the natural organization of the hly locus.  Ref: 9630987 Nat Biotechnol, 1996
